# Does the Species Number of Invasive Plants Regulate the Intensity of Interspecific Interactions Among Multiple Plants Under Different Invasion Scenarios?

**DOI:** 10.3390/plants14172767

**Published:** 2025-09-04

**Authors:** Yizhuo Du, Yingsheng Liu, Xiaoxuan Geng, Yulong Zhang, Congyan Wang, Daolin Du

**Affiliations:** 1 School of Environment and Safety Engineering, Jiangsu University, Zhenjiang 212013, China; 2212409006@stmail.ujs.edu.cn (Y.D.); 2222409004@stmail.ujs.edu.cn (Y.L.); gengxiaoxuan2025@163.com (X.G.); zyl@stmail.ujs.edu.cn (Y.Z.); 2 Key Laboratory of Ocean Space Resource Management Technology, Marine Academy of Zhejiang Province, Hangzhou 310012, China; 3 Jiangsu Collaborative Innovation Center of Technology and Material of Water Treatment, Suzhou University of Science and Technology, Suzhou 215009, China; 4 Jingjiang College, Jiangsu University, Zhenjiang 212013, China; ddl@ujs.edu.cn

**Keywords:** community invasibility, co-invasion, invasion intensity, plant taxonomic diversity, relative coverage

## Abstract

Multiple invasive plants (IPS) can coexist in the same community. The intensity of interspecific interactions among multiple plants may progressively alter with the differences in the species number of IPS (*S_i_*) under different invasion scenarios. However, the correlation between plant taxonomic diversity, *S_i_*, the invasion intensity of IPS, the community invasibility and the intensity of interspecific interactions among multiple plants under different invasion scenarios remains unclear. This study aims to estimate the differences in the intensity of interspecific interactions among multiple plants, the taxonomic diversity of plants, the invasion intensity of IPS and the invasibility of the plant community under different invasion scenarios along a gradient of *S_i_*. This study used a comparative field survey method in four cities in Jiangsu (including Lianyungang, Yancheng, Nantong and Zhenjiang), China. The species number of plants and plant richness decreased under the mono-invasion achieved by one IP compared to the uninvaded communities. Plant taxonomic diversity was negatively associated with the invasion intensity of IPS and the community invasibility. Plant taxonomic diversity was positively associated with *S_i_*. The intensity of interspecific interactions among multiple plants decreased across all invasion scenarios. The intensity of interspecific interactions among multiple plants showed a significant positive association with the ratio of the max and min relative coverage of all plants, but a significant negative association with plant evenness. Therefore, the ratio of the max and min relative coverage of all plants and plant evenness may be the main factor regulating the intensity of interspecific interactions among multiple plants under different invasion scenarios, rather than *S_i_*.

## 1. Introduction

Invasive plants (IPS) can significantly alter the structure and function of native habitats. Specifically, the invasion of these IPS can significantly reduce the diversity of native plants but significantly increase the community invasibility in the invaded habitats [1,2,3]. Therefore, explaining the mechanisms behind the successful invasion of these IPS has become one of the major key scientific issues in the field of invasion ecology in recent decades [1,4,5].

It is worth noting that multiple IPS can coexist in a given plant community [1,6,7]. More importantly, one IP can increase the chances of another IP successfully invading via the facilitative interactions by changing relevant environmental conditions, such as altering the competitive relationship and relative advantage comparison between IPS and native plants, leading to the co-invasion of two or even more IPS in the same habitat [8,9,10]. Nevertheless, under different invasion scenarios, the differences in the structure and function of native habitats may gradually fluctuate with the differences in the species number of IPS (*S_i_*). In particular, plant taxonomic diversity, the invasion intensity of IPS and the community invasibility may progressively alter with the differences in *S_i_* under different invasion scenarios, mainly due to the difference in the intensity of interspecific interactions among multiple plants, especially those between IPS and native plants. Nevertheless, it is presently uncertain whether the correlation between plant taxonomic diversity, *S_i_*, the invasion intensity of IPS, the community invasibility and the intensity of interspecific interactions among multiple plants under different invasion scenarios along a gradient of *S_i_*. Therefore, it is crucial to determine the influences of plant taxonomic diversity, *S_i_*, the invasion intensity of IPS and the community invasibility on the intensity of interspecific interactions among multiple plants under different invasion scenarios along a gradient of *S_i_*. However, the research progress in this field is limited.

This study aims to estimate the differences in the intensity of interspecific interactions among multiple plants, plant taxonomic diversity, the invasion intensity of IPS and the community invasibility under different invasion scenarios along a gradient of *S_i_*. Furthermore, the correlation between plant taxonomic diversity, *S_i_*, the invasion intensity of IPS, the community invasibility and the intensity of interspecific interactions among multiple plants under different invasion scenarios were assessed. In addition, the influences of plant taxonomic diversity, *S_i_*, the invasion intensity of IPS and the community invasibility on the intensity of interspecific interactions among multiple plants under different invasion scenarios were also evaluated. This study used a comparative field survey method in four cities in Jiangsu (including Lianyungang, Yancheng, Nantong and Zhenjiang), China. The measured plant communities, including the mono-invasion achieved by different IPS and the co-invasion achieved by two and three IPS, were compared to the uninvaded communities.

The current study examines the following hypotheses: (1) the invasion intensity of IPS, the community invasibility and the intensity of interspecific interactions among multiple plants may increase progressively as *S_i_* increases, but plant taxonomic diversity may decrease gradually as *S_i_* increases; (2) the intensity of interspecific interactions among multiple plants may be mainly under the influence of *S_i_*.

## 2. Materials and Methods

### 2.1. Study Design

The plant communities in four cities in Jiangsu (including Lianyungang, Yancheng, Nantong and Zhenjiang), China, were examined from May 2024 to July 2025. The name and the corresponding information about the survey points are shown in Table S1. The principal reason for taking multiple cities as the samples in this study is to minimize the influence of geographical location on the results. All studied plant communities are herbaceous without trees or shrubs. The species composition, source area and life form of IPS in the surveyed plant plots are defined in Table S2. The foremost reason for taking multiple co-invasive IPS of the samples in this study is to minimize the influence of the species of IPS on the results.

In this study, four types of plant plots (size: 1 m × 1 m) were surveyed:

(1) The uninvaded plots (control; no invasion by any IPS) (*n* = 92);
(2) The mono-invasion achieved by one IP (*n* = 827);
(3) The co-invasion achieved by two IPS (*n* = 280);
(4) The co-invasion achieved by three IPS (*n* = 71).

Plant plots with the same invasion scenario were separated by more than 20 m.

The species number of plants (including IPS and native plants) and the relative coverage per plant species were verified for all examined plant plots.

### 2.2. Determination of the Intensity of Interspecific Interactions Among Multiple Plants

To measure the intensity of interspecific interactions among multiple plants in the plant community, the intensity index of interspecific interactions (*IIII*) among multiple plants was estimated as follows:

$$IIII=\sum_{i\;=1}^{S}\;\frac{{RC}_{i}}{{RC}_{j}}\;\times \frac{1}{S^{2}}$$

where *S* is the species number of plants in a given plot. *RC_i_* and *RC_j_* are the relative coverages of plant *i* and *j* in a given plot, respectively. The basic principle of designing this formula is as follows: since the interspecific interactions among two plants are mutual, *IIII* in a given plot is calculated as the ratio of the sum of the relative coverages of any two plant species to the square of the number of plant species in a given plot. The relative coverage of one plant species was calculated as the ratio of its coverage to the total coverage of all plant species in a given plot. The coverage was determined by the vertically projected area of the plant canopy to a large extent [11,12,13]. In particular, the plots with a higher value of *IIII* have a stronger intensity of interspecific interaction among multiple plants in comparison with those with a lower value of *IIII*.

The method for determining *IIII* described in this study was defined by the authors of the present study.

### 2.3. Determination of Plant Taxonomic Diversity, IPS’ Invasion Intensity and the Community Invasibility

Plant taxonomic diversity for all evaluated plant plots was examined using the following: Shannon’s diversity [14], Simpson’s dominance [15], Pielou’s evenness [16] and Margalef’s richness [17] because these indices are often used to measure plant taxonomic diversity.

The invasion intensity index was appraised to assess IPS’ invasion intensity of all invaded plant plots [18].

The community invasibility index was analyzed to quantify the community invasibility of all invaded plant plots [18].

The determination methods for the appraised indices of plant taxonomic diversity, IPS’ invasion intensity and the community invasibility are labeled in Table S3.

### 2.4. Statistical Analysis

Deviations from normality and homogeneity of the raw data of the assessed variances were assessed via the Shapiro–Wilk’s test and Bartlett’s test, respectively, and the assumptions of the anova model were met.

Differences in the values of the appraised variances under different invasion scenarios were evaluated by using the one-way analysis of variance (ANOVA) with Tukey’s test.

Correlation between the measured variances under different invasion scenarios was estimated by using the correlation analysis according to the values of the Pearson product-moment correlation coefficient. Bonferroni corrections were also applied to correct the *p* values to decrease the occurrence probability of type I error.

Influences of *S_i_* on the measured variances and influences of the evaluated variances on *IIII* under different invasion scenarios were quantified by using the path analysis according to the values of the path coefficient (i.e., the standardized regression coefficient).

Statistical analyses were performed using SPSS Statistics 26.0 (IBM, Inc., Armonk, NY, USA).

## 3. Results

### 3.1. Differences in the Measured Indices Under Different Invasion Scenarios

**Figure 1:**
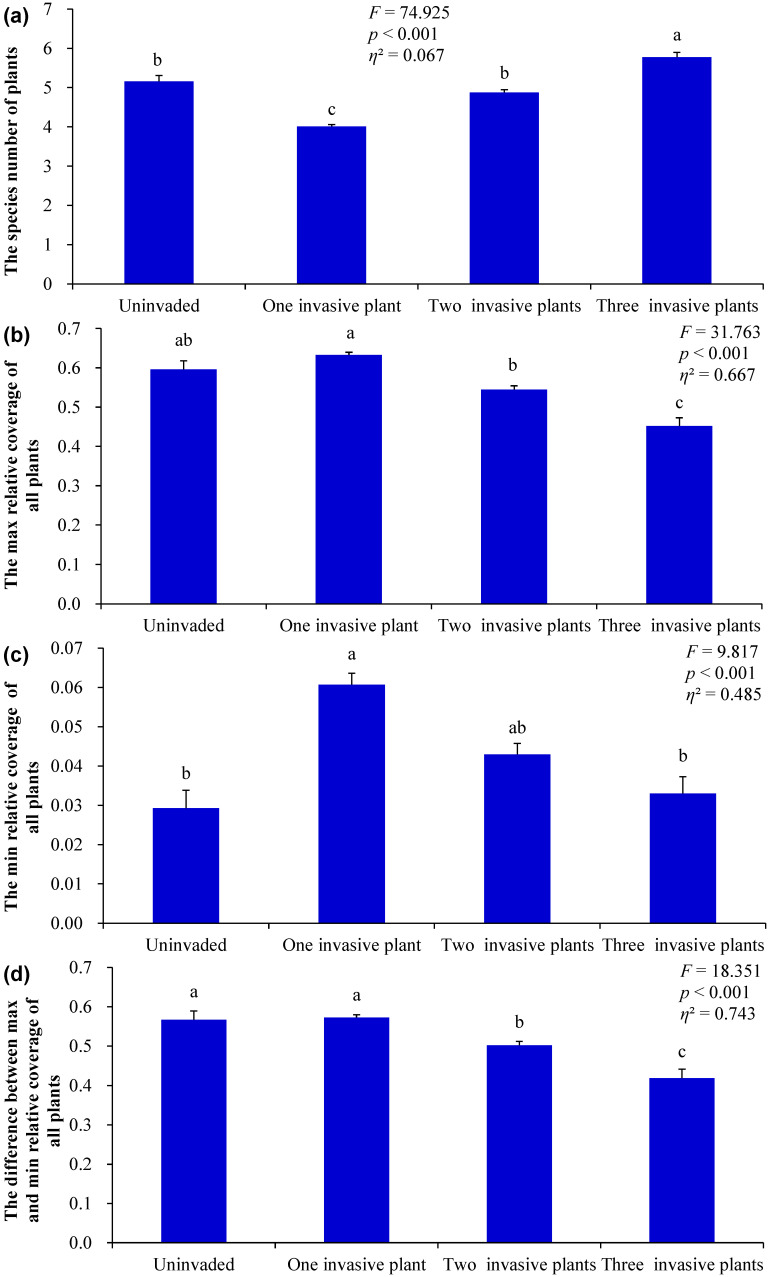

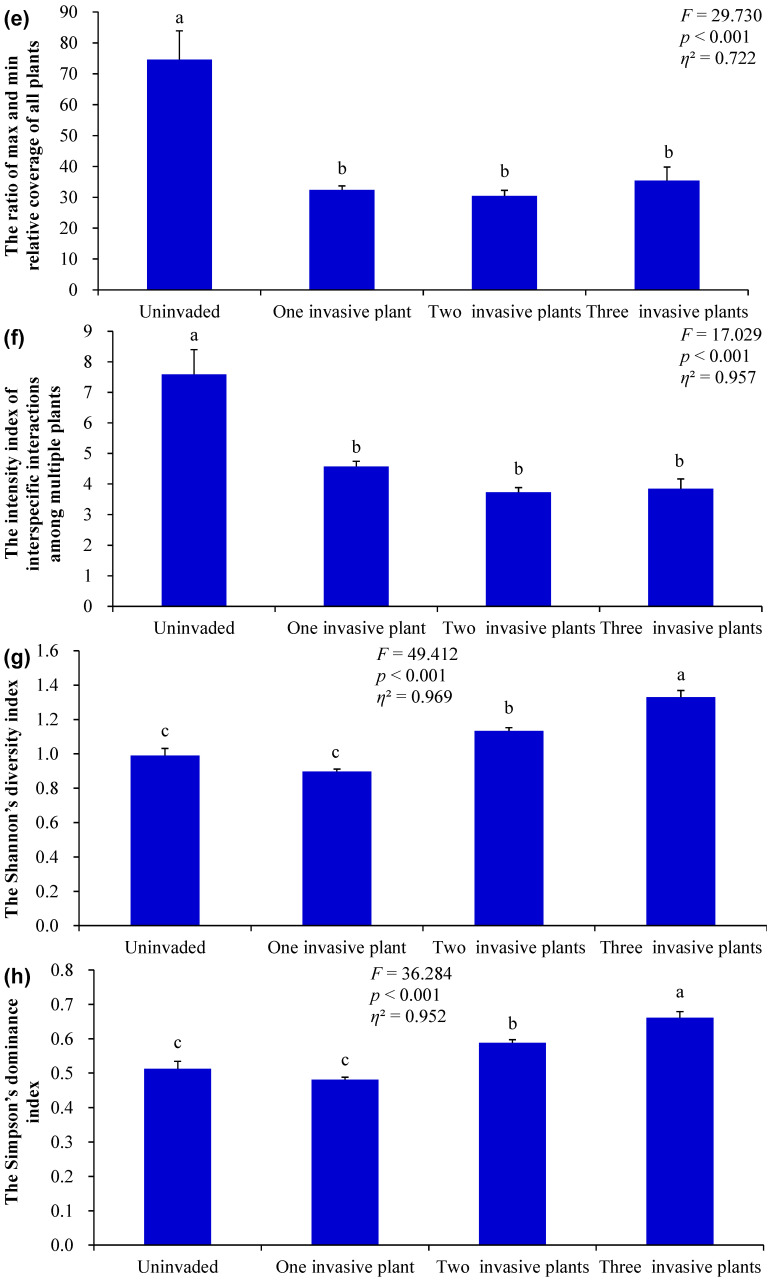

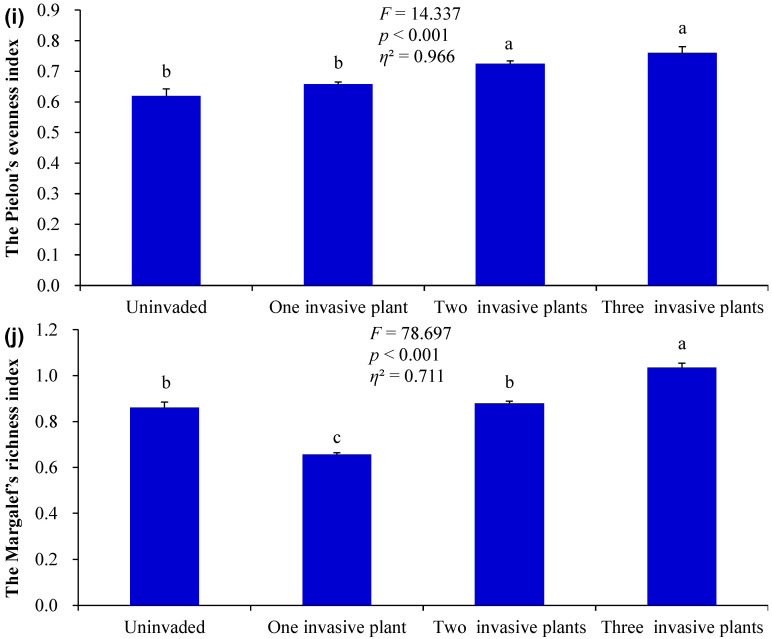
Differences in the measured indices under different invasion scenarios ((**a**), the species number of plants; (**b**), the max relative coverage of all plants; (**c**), the min relative coverage of all plants; (**d**), the difference between max and min relative coverage of all plants; (**e**), the ratio of max and min relative coverage of all plants; (**f**), the intensity index of interspecific interactions among multiple plants; (**g**), the Shannon’s diversity index; (**h**), the Simpson’s dominance index; (**i**), the Pielou’s evenness index; (**j**), the Margalef’s richness index). Data (mean and standard error) with different lowercase letters indicate statistically significant differences at 0.05 probability.

Compared to the uninvaded communities, the mono-invasion achieved by one IP decreased the species number of plants, the ratio of the max and min relative coverage of all plants, *IIII* and the Margalef’s richness index (*p* < 0.05; Figure 1a,e,f,j), whereas it increased the min relative coverage of all plants (*p* < 0.05; Figure 1c).

Compared to the uninvaded communities, the co-invasion achieved by two IPS decreased the difference between the max and min relative coverage of all plants, the ratio of the max and min relative coverage of all plants and *IIII* (*p* < 0.05; Figure 1d–f), whereas it increased the Shannon’s diversity index, the Simpson’s dominance index and the Pielou’s evenness index (*p* < 0.05; Figure 1g–i).

Compared to the uninvaded communities, the co-invasion achieved by three IPS increased the species number of plants and all estimated plant taxonomic diversity indices (*p* < 0.05; Figure 1a,g–j), whereas it decreased the max relative coverage of all plants, the difference between the max and min relative coverage of all plants, and the ratio of the max and min relative coverage of all plants and *IIII* (*p* < 0.05; Figure 1b,d–f).

In invaded communities, the species number of plants, the Shannon’s diversity index, the Simpson’s dominance index and the Margalef’s richness index increased with *S_i_* (*p* < 0.05; Figure 1a,g,h,j); the max relative coverage of all plants and the difference between the max and min relative coverage of all plants decreased with *S_i_* (*p* < 0.05; Figure 1b,d).

Compared to the mono-invasion achieved by one IP, the co-invasion achieved by two and three IPS increased Pielou’s evenness index (*p* < 0.05; Figure 1i); the co-invasion achieved by three IPS decreased the min relative coverage of all plants (*p* < 0.05; Figure 1c).

**Figure 2:**
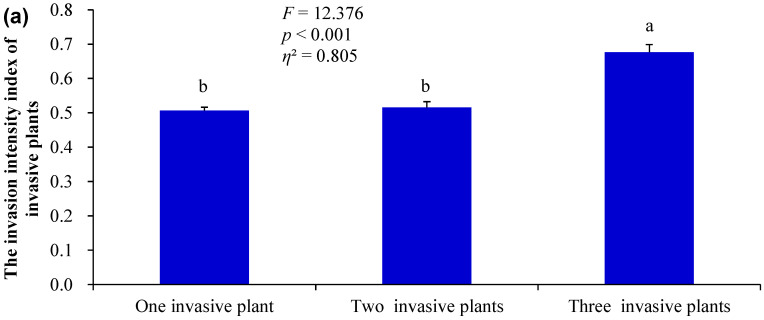

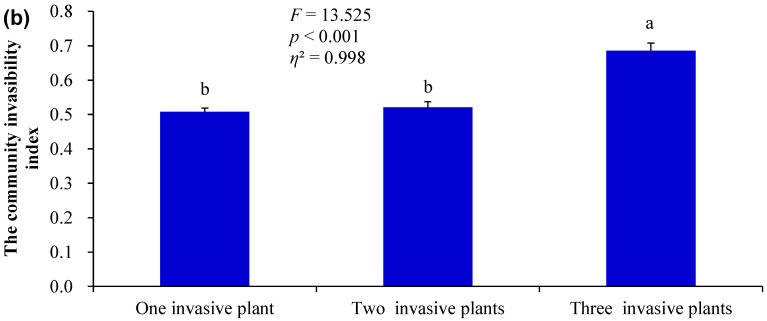
Differences in the invasion intensity index of invasive plants (**a**) and the community invasibility index (**b**) under different invasion scenarios. Data (mean and standard error) with different lowercase letters indicate statistically significant differences at 0.05 probability.

In invaded communities, the values of the invasion intensity index of IPS and the community invasibility index under the co-invasion achieved by three IPS were higher than those under the mono-invasion achieved by one IP and the co-invasion achieved by two IPS (*p* < 0.05; Figure 2a,b).

### 3.2. Correlation Between the Measured Indices Under Different Invasion Scenarios

The species number of plants was positively correlated with the ratio of the max and min relative coverage of all plants, all estimated plant taxonomic diversity indices and *S_i_* (*p* < 0.001; Table 1). The species number of plants was negatively associated with the max and min relative coverage of all plants, the difference between the max and min relative coverage of all plants, the invasion intensity index of IPS and the community invasibility index (*p* < 0.001; Table 1).

*S_i_* was positively correlated with the Shannon’s diversity index, the Simpson’s dominance index, the Margalef’s richness index, the invasion intensity index of IPS and the community invasibility index (*p* < 0.001; Table 1). *S_i_* was negatively associated with the max and min relative coverage of all plants as well as the difference between the max and min relative coverage of all plants (*p* < 0.001; Table 1).

The invasion intensity index of IPS was positively correlated with the community invasibility index (*p* < 0.001; Table 1). The invasion intensity index of IPS and the community invasibility index were positively linked to the max relative coverage of all plants, the difference between the max and min relative coverage of all plants and the ratio of the max and min relative coverage of all plants (*p* < 0.001; Table 1). The invasion intensity index of IPS and the community invasibility index were negatively associated with the Shannon’s diversity index, the Simpson’s dominance index and the Margalef’s richness index (*p* < 0.001; Table 1).

*IIII* was positively correlated with the max relative coverage of all plants, the difference between the max and min relative coverage of all plants, the ratio of the max and min relative coverage of all plants, the invasion intensity index of IPS and the community invasibility index (*p* < 0.001; Table 1). *IIII* was negatively associated with the min relative coverage of all plants and all estimated plant taxonomic diversity indices (*p* < 0.001; Table 1).

### 3.3. Influences of S_i_ on the Measured Variances as Well as Influences of the Measured Variances on IIII Under Different Invasion Scenarios

**Figure 3:**
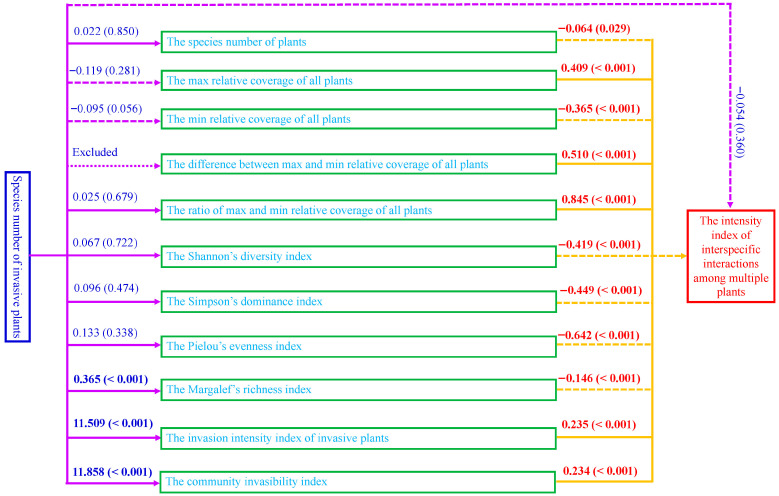
Schematic diagram of influences of the species number of invasive plants (dark blue numbers) on the measured variances and influences of the measured variances (fuchsia numbers) on the intensity index of interspecific interactions among multiple plants under different invasion scenarios. Positive values (solid lines) mean positive influences, while negative values (dashed lines) mean negative influences. The stronger the influence, the greater the deviation from 0, and vice versa. The *p* value is presented in parentheses. *p* ≤ 0.05 are shown in bold.

The ratio of the max and min relative coverage of all plants activated the max positive influence on *IIII* among the measured variances (*p* < 0.05; Figure 3).

Pielou’s evenness index generated max negative influences on *IIII* among the measured variances (*p* < 0.05; Figure 3).

## 4. Discussion

The mono-invasion achieved by one IP significantly decreased the species number of plants and plant richness in the invaded habitats compared to the uninvaded communities (Figure 1a,j). The finding may be attributed to the fact that IPS has stronger growth competitiveness compared to native plants [2,3,5]. More importantly, the results of the correlation analysis indicated that the invasion intensity of IPS was negatively correlated with plant taxonomic diversity (Table 1). This may mean that as the invasion intensity of IPS progressively upsurges, the growth competitiveness of native plants gradually declines, leading to the extinction of some species of native plants. This may further lead to a decrease in the resistance of native plant communities to IPS’ invasion, while increasing their invasibility. In addition, the results of the correlation analysis showed that the invasion intensity of IPS was positively correlated with the community invasibility, but the community invasibility was negatively correlated with plant taxonomic diversity (Table 1).

In other words, high plant taxonomic diversity generates more stable plant communities that are more resistant to IPS’ invasion but with lower community invasibility [19,20,21]. The main reason for the negative correlation between plant taxonomic diversity and community invasibility may be attributed to the fact that a higher level of plant diversity can increase the efficiency of acquisition of ecological resources, recognized as the complementary resource use, and then decrease the community invasibility, but increase the invasion resistance [19,22,23,24]. The negative correlation between plant taxonomic diversity and the community invasibility may also be attributed to the asynchronism in the relative coverage of different plants, i.e., the reduction in the relative coverage of some plants may be counteracted by the amplification in the relative coverage of other functionally comparable plants [25,26,27]. Consequently, the mono-invasion achieved by one IP can cause a decrease in plant taxonomic diversity in the invaded habitats compared to the uninvaded communities, and plant taxonomic diversity may be negatively associated with the invasion intensity of IPS and the community invasibility.

However, the co-invasion achieved by two and three IPS recruited an increase in plant taxonomic diversity compared to the mono-invasion achieved by one IP (Figure 1a,g–j). In particular, plant taxonomic diversity increases with *S_i_* under different invasion scenarios (Figure 1a,g–j). Furthermore, *S_i_* was positively correlated with plant taxonomic diversity under different invasion scenarios (Table 1). Therefore, plant taxonomic diversity was linked to *S_i_* under different invasion scenarios. This finding is inconsistent with the first hypothesis. The positive effects of *S_i_* on plant taxonomic diversity under different invasion scenarios may be ascribed to the fact that the co-invasion of multiple IPS permits these IPS to act as a positive driver rather than a passenger [19,28,29]. In addition, this phenomenon may be attributable to the antagonistic effects rather than synergistic effects of multiple IPS on the native communities under the co-invasion scenarios compared to their mono-invasion; that is to say, multiple IPS may interfere with each other potentially when they invade together. This phenomenon is named invasion interference. Precisely, the invasion interference between multiple IPS when they invade together can lead to a reduction in their combined effects on the native plant community structure. Additionally, the invasion interference between multiple IPS under co-invasion scenarios has been identified in numerous earlier studies [18,30,31], and has been supposed to be attributed to the robust competitiveness to obtain limited ecological resources, which upsurges from the interspecific and intraspecific competition increasing gradually under different co-invasion scenarios. More importantly, based on the habitat filtering hypothesis [32,33,34], due to natural selection achieved through the environmental factors screening for stress, the ecological niches of plants in the same habitat may be close to each other. Nevertheless, similar ecological niches between multiple plants can cause a strong intensity in the intraspecific and interspecific competition for the acquisition of ecological resources according to the niche differentiation hypothesis and Gause’s hypothesis [35,36,37]. Furthermore, the positive effects of the co-invasion achieved by two and three IPS may also be attributed to the fact that the co-invasion achieved by two and three IPS may trigger a certain degree of disturbance on the native plant community structure. More importantly, according to the intermediate disturbance hypothesis, the appropriate disturbance may maintain a high level of plant diversity [38,39,40].

The intensity of interspecific interactions among multiple plants decreased under IPS’ invasion (Figure 1f). However, there was no significant difference in the intensity of interspecific interactions among multiple plants under different invasion scenarios (Figure 1f). Accordingly, the intensity of interspecific interactions among multiple plants may be decreased under IPS’ invasion, regardless of the invasion scenario. This finding is inconsistent with the first hypothesis. This phenomenon may be attributed to the decreased ratio of the max and min relative coverage of all plants and the increased plant evenness under IPS’ invasion (Figure 1e,i). The results of both the correlation analysis and the path analysis presented that the intensity of interspecific interactions among multiple plants was positively correlated with the ratio of the max and min relative coverage of all plants but was negatively associated with plant evenness (Table 1 and Figure 3). Hence, the result is inconsistent with the second hypothesis. Therefore, compared to the habitats with lower ratios of the max and min relative coverage of all plants (but with higher plant evenness), habitats with higher ratios of the max and min relative coverage of all plants (but with lower plant evenness) have a stronger intensity of interspecific interactions among multiple plants. In other words, once the distribution of different plants becomes more non-uniform, gradually shifting from uniformity in a given habitat, the ratio of the max and min relative coverage of all plants will progressively increase, and the same changes will also occur in the intensity of interspecific interactions among multiple plants. In particular, the intensified interspecific interactions among multiple plants can lead to an increase in the relative coverage of plants with stronger competitive ability for ecological resources, but result in a decrease in the relative coverage or even loss of plants with weaker competitive ability for ecological resources, with gradually increase in the ratio of the max and min relative coverage of all plants but with progressively decrease in plant evenness. This will create a possible situation where the strong become stronger and the weak become weaker.

## 5. Conclusions

This study aimed to determine how the plant community structure affects the intensity of interspecific interactions among multiple plants under different invasion scenarios. The mono-invasion achieved by one IP decreased the species number of plants and plant richness in the invaded habitats compared to the uninvaded communities. Plant taxonomic diversity increased with *S_i_* under different invasion scenarios. The invasion intensity of IPS and the community invasibility were negatively correlated with plant taxonomic diversity. IPS’ invasion, regardless of the invasion scenario, decreased the intensity of interspecific interactions among multiple plants compared to the uninvaded communities. The intensity of interspecific interactions among multiple plants was positively correlated with the ratio of the max and min relative coverage of all plants but was negatively correlated with plant evenness under different invasion scenarios. Thus, the ratio of the max and min relative coverage of all plants and plant evenness may be the main factor regulating the intensity of interspecific interactions among multiple plants under different invasion scenarios, rather than the species number of invasive plants.

## Figures and Tables

**Table 1:** Correlation (*r*, the Pearson’s coefficient) between the measured indices under different invasion scenarios. *p* values equal to or lower than the corrected *p* value (corrected *p* value = 0.05/(13 × 12/2) = 0.000641) by using Bonferroni correction are shown in bold. Abbreviation: *S*, the species number of plants; *MaxRC_i_*, the max relative coverage of all plants; *MinRC_i_*, the min relative coverage of all plants; *IIII*, the intensity index of interspecific interactions among multiple plants; *H’*, the Shannon’s diversity index; *D*, the Simpson’s dominance index; *E_H_*, the Pielou’s evenness index; *F*, the Margalef’s richness index; *III*, the invasion intensity index of invasive plants; *CII*, the community invasibility index; *S_i_*, the species number of invasive plants.

*r*	*S*	*MaxRC_i_*	*MinRC_i_*	Difference Between *MaxRC_i_* and *MinRC_i_*	Ratio of *MaxRC_i_* and *MinRC_i_*	*IIII*	*H’*	*D*	*E_H_*	*F*	*III*	*CII*	*S_i_*
***S***	1	**−0.599**	**−0.474**	**−0.381**	**0.149**	−0.064	**0.787**	**0.661**	**0.303**	**0.936**	**−0.380**	**−0.378**	**0.374**
***MaxRC_i_***		1	−0.022	**0.932**	**0.367**	**0.409**	**−0.928**	**−0.967**	**−0.864**	**−0.617**	**0.481**	**0.478**	**−0.276**
***MinRC_i_***			1	**−0.383**	**−0.436**	**−0.365**	**−0.134**	**−00.015**	**0.390**	**−0.434**	0.033	0.033	**−0.122**
**Difference between *MaxRC_i_* and *MinRC_i_***				1	**0.497**	**0.510**	**−0.808**	**−0.888**	**−0.940**	**−0.412**	**0.432**	**0.430**	**−0.211**
**Ratio of *MaxRC_i_* and *MinRC_i_***					1	**0.845**	**−0.302**	**−0.356**	**−0.569**	00.041	**0.189**	**0.189**	<0.001
***IIII***						**1**	**−0.419**	**−0.449**	**−0.642**	**−0.146**	**0.235**	**0.234**	−0.080
***H’***							1	**0.955**	**0.794**	**0.789**	**−0.493**	**−0.490**	**0.335**
***D***								1	**0.865**	**0.677**	**−0.505**	**−0.502**	**0.292**
***E_H_***									1	0.345	−0.424	−0.422	0.170
***F***										1	**−0.387**	**−0.384**	**0.395**
***III***											1	**1.000**	**0.110**
***CII***												1	**0.118**
***S_i_***													1

## Data Availability

The data presented in this study are available on request from the corresponding author. The data are not publicly available due to privacy and ethical restrictions.

## Supplementary Materials

The following supporting information can be downloaded at https://www.mdpi.com/article/10.3390/plants14172767/s1, Table S1: The name and the corresponding information about the survey sites in this study; Table S2: Information about the species composition, source area and life form of invasive plants; Table S3: The determination methods and the corresponding references for the analyzed variables in this study.
