# Supplementary material for: Does the Species Number of Invasive Plants Regulate the Intensity of Interspecific Interactions Among Multiple Plants Under Different Invasion Scenarios?
Source: Plants (Basel). 2025 Sep 4;14(17):2767. doi: 10.3390/plants14172767 (PMC12430376; doi:10.3390/plants14172767)
Supplement: Supplementary file 1 [file plants-14-02767-s001.zip › plants-3818052-supplementary.pdf]

**Table S1** The name and the corresponding information about the survey sites in this study.

| **Chinese name of province** | **English name of province** | **Chinese name of city** | **English name of city** | **Chinese name of climate type** | **English name of climate type** | **Chinese name of sampling point** | **English name of sampling point** | **Latitude and longitude** |
|---|---|---|---|---|---|---|---|---|
| 江苏 | Jiangsu | 连云港 | Lianyungang | 温带季风气候 | Temperate monsoon climate | 西墅大沙湾 | Xishudashawan Park | 34.762° N, 119.330−119.331° E |
| | | | | | | 在海一方公园 | Zaihaiyifang Park | 34.757−34.759° N, 119.363−119.367° E |
| | | | | | | 凰窝风景区 | Huangwo Scenic Area | 34.717° N, 119.472−119.473° E |
| | | | | | | 燕板线附近 | Near the Yanbanxian Road | 34.497−34.512° N, 119.631−119.765° E |
| | | | | | | 临海公路附近 | Near the Coastal highway | 34.493° N, 119.629−119.630° E |
| | | 盐城 | Yancheng | | Transition Zone from | 新三线附近 | Near the Xinsanxian Road | 34.423−34.441° N, 119.896−120.006° E |
| | | | | | | 新滩路附近 | Near the Xintan Road | 34.339° N, 120.181° E |

| | | | | | | |
|---|---|---|---|---|---|---|
| | | 北亚热带向南暖温带过渡区 | North Subtropical to South Warm Temperate Zone | 古黄河入海口 | The Ancient Yellow River Estuary | 34.257−34.260° N, 120.270−120.275° E |
| | | | | 国华二期北风电场附近 | Near the Guohua Phase II North Wind Power Plant | 32.949−32.950° N, 120.894−120.895° E |
| | | | | 黄海森林生态旅游区 | Huanghai Forest Ecotourism Area | 32.874−32.875° N, 120.905−120.906° E |
| 南通 | Nantong | 北亚热带海洋性季风气候 | North subtropical oceanic monsoon climate | 滨海南路附近 | Near the Binhai South Road | 32.612−32.613° N, 120.950° E |
| | | | | 堤顶路附近 | Near the Dike Top Road | 32.592° N, 120.979° E |
| | | | | 金蛤大道附近 | Near the Jinhe Avenue | 32.577° N, 121.004° E |
| | | | | 海印寺附近 | Near the Haiyin Temple | 32.559° N, 121.041−121.042° E |
| | | | | 通海六路附近 | Near the Tonghai Sixth Road | 32.540° N, 121.112° E |

| | | | | | | |
|---|---|---|---|---|---|---|
| | | | | 海上环港风电场附近 | Near the Offshore Port Wind Power Plant | 32.512−32.513° N, 121.170−121.175° E |
| | | | | 环东海堤线附近 | Near the East China Sea Embankment Line | 32.490° N, 121.163° E |
| 镇江 | Zhenjiang | 北亚热带季风气候 | North subtropical monsoon climate | 滨江路附近 | Near the Binjiang Road | 32.238−32.239° N, 119.502−119.503° E |
| | | | | 长江堤岸 | Yangtze River Embankment | 32.209−32.212° N, 119.531−119.532° E |
| | | | | 横山凹 | Hengshanao | 32.161−32.163° N, 119.529−119.530° E |
| | | | | 大冢岗 | Dazhonggang | 32.157−32.158° N, 119.519−119.520° E |
| | | | | 金润大道附近 | Near Jinrun Avenue | 32.145−32.144° N, 119.519−119.523° E |
| | | | | 前北村附近 | Near the Qianbei Village | 32.112−32.114° N, 119.519° E |

**Table S2** Information about the species composition, source area and life form of invasive plants.

| Chinese name of invasive plants | Latin name of invasive plants | Chinese name of Family | Latin name of Family | Chinese name of Genus | Latin name of Genus | Chinese name of place of origin | English name of place of origin | Chinese name of life form | English name of life form |
|---|---|---|---|---|---|---|---|---|---|
| 白车轴草 | *Trifolium repens* L. | 豆科 | Fabaceae | 车轴草属 | *Trifolium* | 欧洲和北非 | Europe and North Africa | 多年生草本 | Perennial herb |
| 北美车前 | *Plantago virginica* L. | 车前科 | Plantaginaceae | 车前属 | *Plantago* | 北美 | North America | 一年生或二年生草本 | Annual or biennial herb |
| 臭荠 | *Lepidium didymum* L. | 十字花科 | Brassicaceae | 独行菜属 | *Lepidium* | 南美 | South America | 一年生或二年生草本 | Annual or biennial herb |
| 垂序商陆 | *Phytolacca americana* L. | 商陆科 | Phytolaccaceae | 商陆属 | *Phytolacca* | 北美 | North America | 多年生草本 | Perennial herb |

| | | | | | | | | | |
|---|---|---|---|---|---|---|---|---|---|
| 春飞蓬 | *Erigeron philadelphicus* L. | 菊科 | Asteraceae | 飞蓬属 | *Erigeron* | 北美 | North America | 一年生或二年生草本 | Annual or biennial herb |
| 大白花鬼针草 | *Bidens alba* (L.) DC. | 菊科 | Asteraceae | 鬼针草属 | *Bidens* | 北美 | North America | 一年生草本 | Annual herb |
| 鬼针草 | *Bidens pilosa* L. | 菊科 | Asteraceae | 鬼针草属 | *Bidens* | 南美 | South America | 一年生草本 | Annual herb |
| 互花米草 | *Spartina alterniflora* Loisel. | 禾本科 | Poaceae | 米草属 | *Spartina* | 北美 | North America | 多年生草本 | Perennial herb |
| 火炬树 | *Rhus typhina* L. | 漆树科 | Anacardiaceae | 盐麸木属 | *Rhus* | 北美 | North America | 落叶灌木或乔木 | Deciduous shrub or tree |
| 加拿大一枝黄花 | *Solidago canadensis* L. | 菊科 | Asteraceae | 一枝黄花属 | *Solidago* | 北美 | North America | 多年生草本 | Perennial herb |
| 空心莲子草 | *Alternanthera philoxeroides* (Mart.) Griseb. | 苋科 | Amaranthaceae | 莲子草属 | *Alternanthera* | 南美 | South America | 多年生草本 | Perennial herb |
| 苦蘵 | *Physalis angulata* L. | 茄科 | Solanaceae | 洋酸浆属 | *Physalis* | 南美 | South America | 一年生草本 | Annual herb |

| | | | | | | | | | |
|---|---|---|---|---|---|---|---|---|---|
| 美丽月见草 | *Oenothera speciosa* Nutt. | 柳叶菜科 | Onagraceae | 月见草属 | *Oenothera* | 北美 | North America | 多年生草本 | Perennial herb |
| 青葙 | *Celosia argentea* L. | 苋科 | Amaranthaceae | 青葙属 | *Celosia* | 北美 | North America | 一年生草本 | Annual herb |
| 三裂叶薯 | *Ipomoea triloba* L. | 旋花科 | Convolvulaceae | 番薯属 | *Ipomoea* | 南美 | South America | 一年生草本 | Annual herb |
| 苏门白酒草 | *Erigeron sumatrensis* Retz. | 菊科 | Asteraceae | 飞蓬属 | *Erigeron* | 南美 | South America | 一年生或二年生草本 | Annual or biennial herb |
| 田菁 | *Sesbania cannabina* (Retz.) Pers. | 豆科 | Fabaceae | 田菁属 | *Sesbania* | 南亚和澳洲 | South Asia and Australia | 一年生草本 | Annual herb |
| 小蓬草 | *Erigeron canadensis* L. | 菊科 | Asteraceae | 飞蓬属 | *Erigeron* | 北美 | North America | 一年生草本 | Annual herb |
| 野老鹳草 | *Geranium carolinianum* L. | 牻牛儿苗科 | Geraniaceae | 老鹳草属 | *Geranium* | 北美 | North America | 多年生草本 | Perennial herb |
| 野莴苣 | *Lactuca serriola* L. | 菊科 | Asteraceae | 莴苣属 | *Lactuca* | 欧洲和中亚 | Europe and Central Asia | 一年生或二年生草本 | Annual or biennial herb |

| | | | | | | | | | |
|---|---|---|---|---|---|---|---|---|---|
| 一年蓬 | *Erigeron annuus* (L.) Pers. | 菊科 | Asteraceae | 飞蓬属 | *Erigeron* | 北美 | North America | 一年生或二年生草本 | Annual or biennial herb |
| 圆叶牵牛 | *Ipomoea purpurea* (L.) Roth | 旋花科 | Convolvulaceae | 番薯属 | *Ipomoea* | 北美 | North America | 一年生草本 | Annual herb |
| 直立婆婆纳 | *Veronica arvensis* L. | 车前科 | Plantaginaceae | 婆婆纳属 | *Veronica* | 欧洲 | Europe | 一年生草本 | Annual herb |
| 钻叶紫菀 | *Symphyotrichum subulatum* (Michx.) G. L. Nesom | 菊科 | Asteraceae | 联毛紫菀属 | *Symphyotrichum* | 北美 | North America | 一年生草本 | Annual herb |

**Table S3** The determination methods and the corresponding references for the analyzed variables in this study.

| Measured indices | Determination methods | References |
|---|---|---|
| Relative coverage (*RC*) | $$RC = \frac{C_i}{C}$$ where $C_i$ represents the coverage of plant species $i$ and $C$ represents the total coverage of all plant species in one particular plot, respectively. The coverage was determined by the vertically projected area of the plant canopy to a large extent. | [1-3] |
| Shannon's diversity index ($H'$) | $$H' = -\sum_{i=1}^{S} RC_i \ln RC_i$$ where $S$ represents the number of plant species and $RC_i$ represents the relative coverage of plant species $i$ in one particular plot, respectively. | [4] |
| Simpson's dominance index ($D$) | $$D = 1 - \sum_{i=1}^{S} RC^2$$ where $S$ represents the number of plant species and $RC_i$ represents the relative coverage of plant species $i$ in one particular plot, respectively. | [5] |

| | | |
|---|---|---|
| Pielou's evenness index ($E_H$) | $$E_H = \frac{H'}{\ln S}$$ where $S$ represents the number of plant species and $H'$ is Shannon's diversity index in one particular plot, respectively. | [6] |
| Margalef's richness index ($F$) | $$F = \frac{S - 1}{\ln RC}$$ where $S$ represents the number of plant species and $RC$ is the total coverage of all plant species in one particular plot, respectively. | [7] |
| Invasion intensity index ($III$) | $$III = \frac{RC_i}{MaxRC_i}$$ where $RC_i$ represents the relative coverage of invasive plant species $i$ in one invaded quadrat and $MaxRC_i$ is the max value of relative coverage of invasive plant species $i$ among all invaded quadrats, respectively. In particular, invasive plant species in the invaded quadrats with higher values of $III$ possess greater degree of invasion intensity compared to those with lower values of $III$. | [8] |

| | | |
|---|---|---|
| Community invasibility index (*CII*) | $CII = 1 - (MaxRC_i - RC_i)$ where $RC_i$ represents the observed relative coverage of invasive plant species in one invaded quadrat and $MaxRC_i$ is the max relative coverage of invasive plant species among all invaded quadrats, respectively. In particular, the invaded quadrats with higher values of *CII* possess greater degree of invasibility compared to those with lower values of *CII*. | [8] |

**References**


1. Guo, X.; Li, M. Y.; Jiang, S. Y.; Yang, L. Y.; Guo, S. X.; Xing, L. J.; Wang, T., Arbuscular mycorrhizal fungi inoculation exerts weak effects on species- and community-level growth traits for invading or native plants under nitrogen deposition. *Frontiers in Ecology and Evolution* **2023**, *11*, 1152213.
2. Wang, G. N.; Wang, X. F.; Zhang, Y.; Yang, J.; Li, Z. K.; Wu, L. Z.; Wu, J. H.; Wu, N.; Liu, L. X.; Liu, Z. W.; Zhang, M.; Wu, L. Q.; Zhang, G. Y.; Ma, Z. Y., Dynamic characteristics and functional analysis provide new insights into long non-coding RNA responsive to *Verticillium dahliae* infection in *Gossypium hirsutum*. *BMC Plant Biology* **2021**, *21*, 68.
3. Hassan, M. O.; Mohamed, H. Y., Allelopathic interference of the exotic naturalized *Paspalum dilatatum* Poir. threatens diversity of native plants in urban gardens. *Flora* **2020**, *266*, 151593.
4. Shannon, C. E.; Weaver, W., The Mathematical Theory of Communication. University of Illinois Press, Urbana, Illinois. **1949**, 1-117.
5. Simpson, E. H., Measurement of diversity. *Nature* **1949**, *163*, 688.
6. Pielou, E. C., The measurement of diversity in different types of biological collections. *Journal of Theoretical Biology* **1966**, *13*, 131-144.

7. Margalef, R., Diversidad de especies en las comunidades naturales. Barcelona: Publicaciones del Instituto de Biologia Aplicada. **1951**, *6*, 59-72.

8. Wang, C. Y.; Wei, M.; Wang, S.; Wu, B. D.; Cheng, H. Y., *Erigeron annuus* (L.) Pers. and *Solidago canadensis* L. antagonistically affect community stability and community invasibility under the co-invasion condition. *Science of the Total Environment* **2020**, *716*, 137128.
